# Recent advances in the understanding and management of hepatorenal syndrome

**DOI:** 10.12703/r/10-48

**Published:** 2021-05-21

**Authors:** Benedikt Simbrunner, Michael Trauner, Thomas Reiberger, Mattias Mandorfer

**Affiliations:** 1 Division of Gastroenterology and Hepatology, Department of Internal Medicine III, Medical University of Vienna, Waehringer Guertel 18-20, 1090 Vienna, Austria; 2 Vienna Hepatic Hemodynamic Lab, Medical University of Vienna, Waehringer Guertel 18-20, 1090 Vienna, Austria

**Keywords:** cirrhosis, portal hypertension, ascites, acute kidney injury, renal impairment

## Abstract

Renal dysfunction occurs frequently in hospitalized patients with advanced chronic liver disease (ACLD)/cirrhosis and has profound prognostic implications. In ACLD patients with ascites, hepatorenal syndrome (HRS) may result from circulatory dysfunction that leads to reduced kidney perfusion and glomerular filtration rate (in the absence of structural kidney damage). The traditional subclassification of HRS has recently been replaced by acute kidney injury (AKI) type of HRS (HRS-AKI) and non-AKI type of HRS (HRS-NAKI), replacing the terms “HRS type 1” and “HRS type 2”, respectively. Importantly, the concept of absolute serum creatinine (sCr) cutoffs for diagnosing HRS was partly abandoned and short term sCr dynamics now may suffice for AKI diagnosis, which facilitates early treatment initiation that may prevent the progression to HRS-AKI or increase the chances of AKI/HRS-AKI reversal. Recent randomized controlled trials have established (a) the efficacy of (long-term) albumin in the prevention of complications of ascites (including HRS-AKI), (b) the benefits of transjugular intrahepatic portosystemic shunt placement in patients with recurrent ascites, and (c) the superiority of terlipressin over noradrenaline for the treatment of HRS-AKI in the context of acute-on-chronic liver failure. This review article aims to summarize recent advances in the understanding and management of HRS.

## Key points

 •    Current diagnostic criteria for acute kidney injury (AKI) are based on changes in serum creatinine, thereby facilitating its early diagnosis, which may prevent progression to the hepatorenal syndrome type of AKI (HRS-AKI) and ensure timely treatment with albumin and vasopressors.
 •    Current data suggest that terlipressin should be preferred over noradrenalin for the treatment of HRS-AKI in the setting of acute-on-chronic liver failure.
 •    Patients with uncomplicated ascites may benefit from the long-term administration of albumin given that renal dysfunction and HRS-AKI presumably are prevented by modulation of systemic inflammation.
 •    Transjugular intrahepatic portosystemic shunt (TIPS) improves hepatic hemodynamics and reduces the risks of HRS-AKI and HRS-NAKI (formerly known as HRS type 2) in patients with recurrent ascites.

## Introduction

Renal dysfunction represents an important complication of advanced chronic liver disease (ACLD)/cirrhosis and is associated with substantially increased morbidity and mortality. The deterioration of kidney function in patients with ACLD has been subdivided into acute kidney injury (AKI), chronic kidney disease (CKD), and hepatorenal syndrome (HRS). Whereas AKI and CKD may occur under similar circumstances as in the general population, HRS is considered a distinct feature of ACLD patients with ascites, is caused by a functional circulatory impairment that is not fully counterbalanced by compensatory mechanisms, and ultimately results in decreased glomerular filtration rate (GFR). In recent years, several definition criteria and (sub)classifications of renal impairment in ACLD have been proposed, mostly aiming to reduce the threshold for diagnosis and treatment. This review aims to provide an overview of the most recent classification of renal dysfunction in ACLD (with a particular focus on HRS), the state-of-the-art therapeutic management, and recent studies providing important implications for an optimized management of HRS.

## Literature research

We screened the latest HRS guidelines of relevant societies, such as the International Club of Ascites (ICA) and the European Association for the Study of the Liver (EASL). Furthermore, a literature search on PubMed was performed by using the keywords “hepatorenal syndrome” and “HRS AND cirrhosis”, prioritizing randomized controlled trials (RCTs), meta-analyses, and observational studies relevant for the recent refinement and adaptions in the HRS definition. Importantly, we have put an emphasis on studies published after latest consensus statements and guidelines by the ICA (2015) and EASL (2018). We considered peer-reviewed studies in English and recently published abstracts from RCTs. Finally, we screened ClinicalTrials.gov for ongoing RCTs by using the search term “hepatorenal syndrome”.

## Definition of renal impairment in advanced chronic liver disease 

### Common definitions of renal impairment

The definition of renal impairment in ACLD has continually evolved during the past decades, usually reflecting the definitions used for acute or chronic kidney disease (or both) in the general population. These definitions rely mostly on serum creatinine (sCr) for the estimation of GFR considering the limitations of sCr as a biomarker in the setting of ACLD^1^. However, owing to its broad availability, sCr is still used to define and quantify kidney dysfunction in ACLD.

First, the Risk, Injury, Failure, Loss of kidney function, and End-stage kidney disease (RIFLE) criteria (Acute Dialysis Quality Initiative Group, 2002) classified acute kidney dysfunction severity on the basis of sCr, GFR, or urine output (or a combination of these)^2^. Further development of this classification (Acute Kidney Injury Network [AKIN], 2005) led to an additional definition criterion of AKI, namely an increase in sCr of 0.3 mg/dL within 48 hours^3^. This decision was based on multiple studies providing evidence for a significant impact of small short-term sCr increases on prognosis^4–6^. Finally, the AKI definition for the general population was refined as an sCr increase of 0.3 mg/dL within 48 hours, an sCr increase of 50% of baseline within 7 days, or urine output of less than 0.5 mL/kg per hour for more than 6 hours (Kidney Disease: Improving Global Outcomes [KDIGO] organization, 2012)^7^.

### Advances in the definition of acute kidney injury in advanced chronic liver disease

The criteria and terminology for AKI and HRS in patients with ACLD have evolved over time. A first consensus agreement (ICA, 1990) defined acute renal failure as an sCr increase by 50% from baseline to an sCr of at least 1.5 mg/dL^8^. The subsequent AKI definition based on AKIN criteria (see above) displayed improved prognostic accuracy for complications and mortality in multiple studies in patients with ACLD^9–15^ and thus was incorporated into ICA consensus recommendations (2015)^16^. Importantly, the ICA also suggested that baseline sCr values within the last 3 months may be considered if no baseline sCr within 7 days is available.

Finally, three AKI stages with additional substages were defined: (a) an sCr increase of at least 0.3 mg/dL within 48 hours or sCr increase by 1.5- to 2-fold from baseline denotes stage 1 (stage 1a** with sCr of less than 1.5 mg/dL and 1b with sCr of at least 1.5 mg/dL), (b) an sCr increase by 2- to 3-fold from baseline defines stage 2, and (c) an sCr increase at least 3-fold from baseline, an sCr value of at least 4 mg/dL in combination with an acute increase by at least 0.3 mg/dL, or initiation of renal replacement therapy (RRT) defines stage 3^16^. Of note, owing to concerns that sodium retention^17^ and the frequent use of diuretics may interfere with this measure^16^, the ICA recommendations did not include reduced urine output as a criterion for defining AKI in ACLD. However, a panel of experts (2019) suggested that this criterion be reinstated in a recent position paper^18^ on the basis of subsequent findings that reduced urine output still has prognostic value^19^.

### Advances in the definition of hepatorenal syndrome in advanced chronic liver disease

The ICA (1996) defined HRS as a functional deterioration of kidney function characterized by pronounced circulatory dysfunction in patients with ACLD and portal hypertension. Type 1 was diagnosed in case of a 2-fold increase of sCr to at least 2.5 mg/dL or a decrease of creatinine clearance by 50% to less than 20 mL/min within 2 weeks. Conversely, cases of renal failure with an sCr of at least 1.5 mg/dL not meeting these criteria were named HRS type 2^8^. An update of the ICA criteria (2007) stated that active bacterial infection—in the absence of shock—is not an exclusion criterion for HRS diagnosis and recommended the use of albumin over saline for plasma expansion^20^. Both the old and more recent ICA consensus recommendations set common prerequisites for HRS: Cirrhosis with ascites, absence of shock, no current or recent intake of nephrotoxic drugs, and no evidence of structural kidney damage (discussed in the ‘Differential diagnosis and novel biomarkers’ section below).

**Figure 1.**
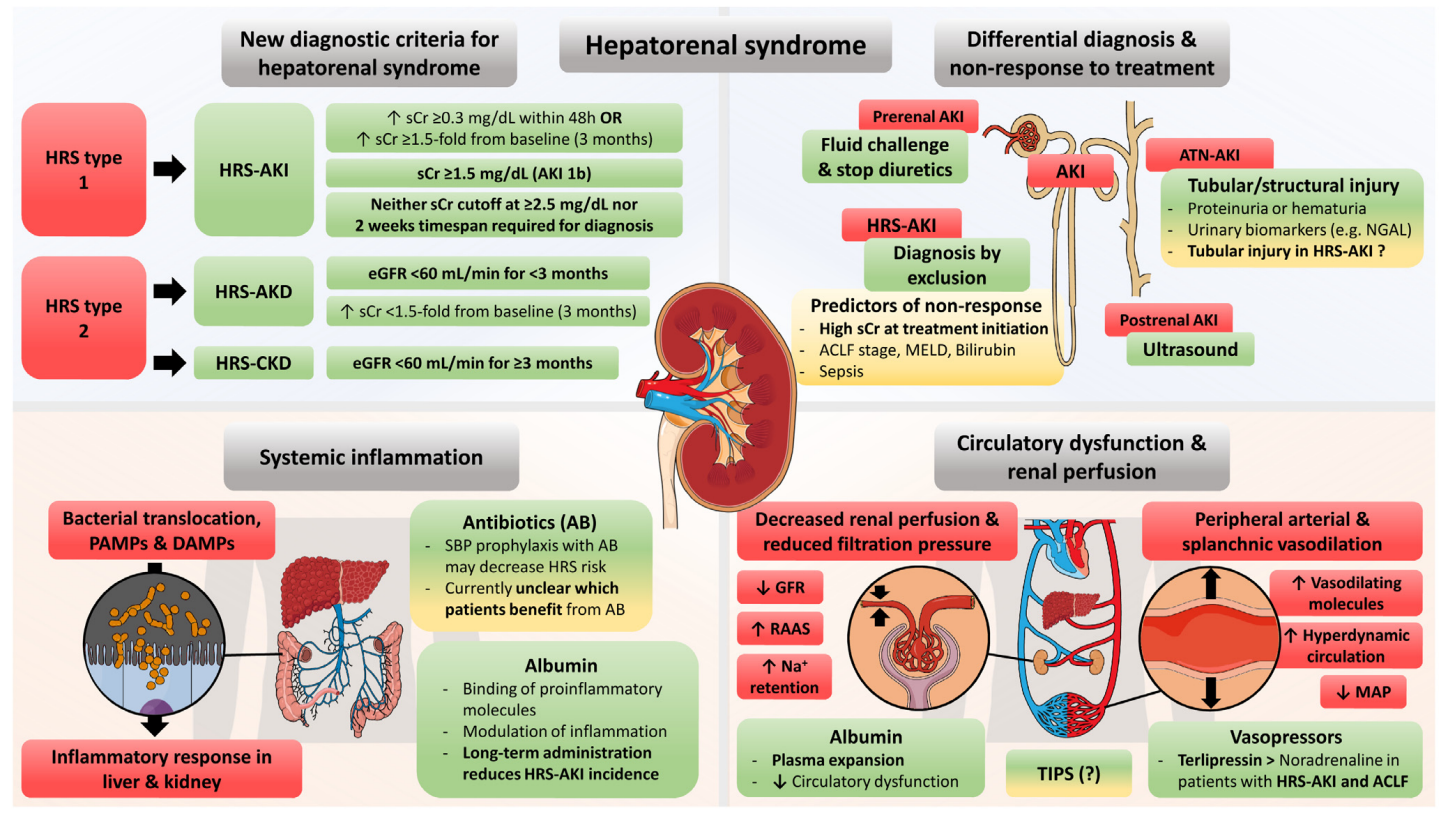
Graphical abstract of advances in understanding and managing hepatorenal syndrome (HRS) in advanced chronic liver disease. ACLF, acute-on-chronic liver failure; AKD, acute kidney disease; AKI, acute kidney injury; ATN, acute tubular necrosis; CKD, chronic kidney disease; DAMP, danger-associated molecular pattern; eGFR, estimated glomerular filtration rate; GFR, glomerular filtration rate; MAP, mean arterial pressure; MELD, model for end-stage liver disease; NGAL, neutrophil gelatinase-associated lipocalin; PAMP, pathogen-associated molecular pattern; RAAS, renin–angiotensin–aldosterone system; SBP, spontaneous bacterial peritonitis; sCr, serum creatinine; TIPS, transjugular intrahepatic portosystemic shunting.

**Figure 2.**
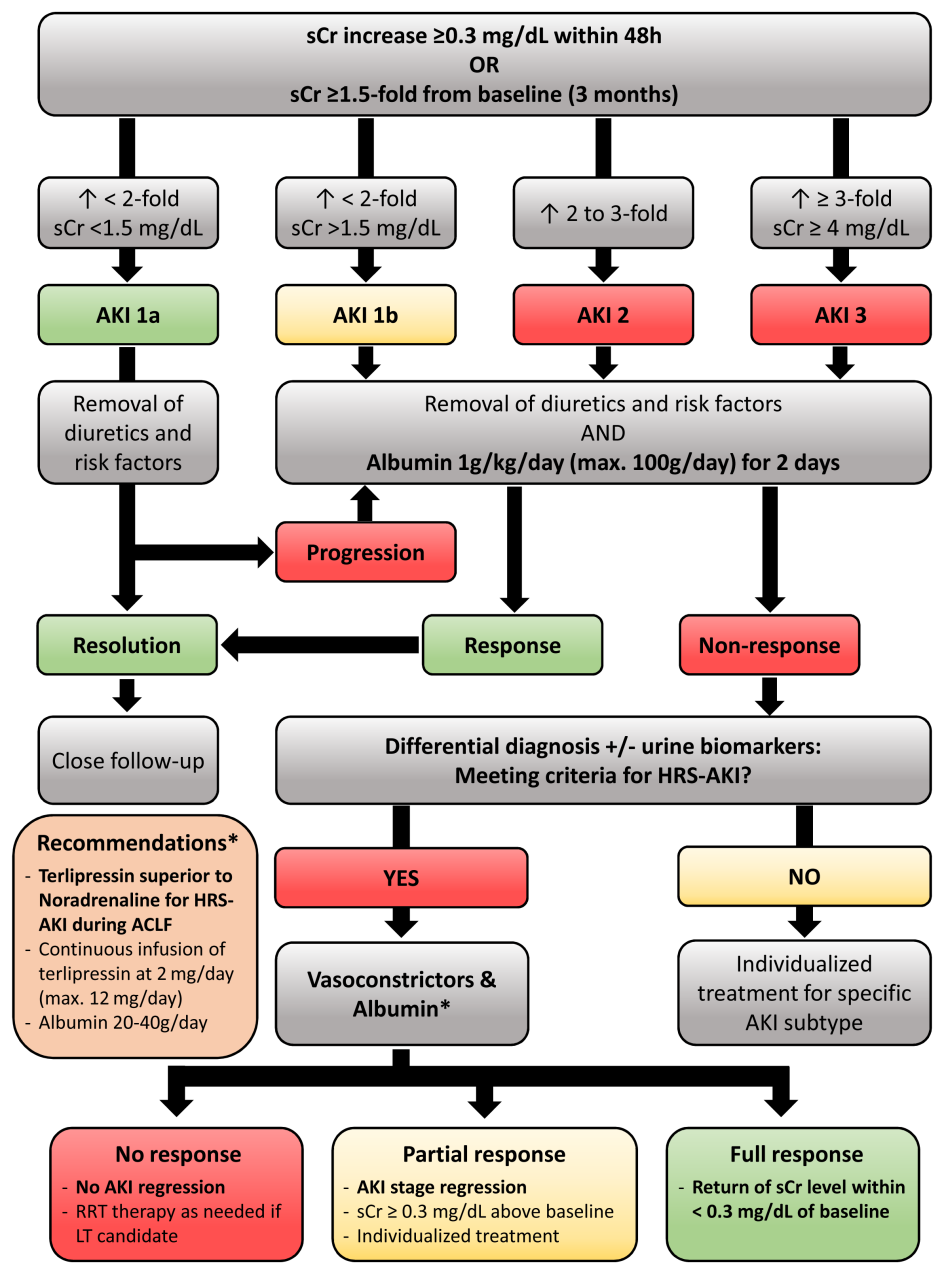
Diagnostic and therapeutic algorithm for identification and management of acute kidney injury (AKI) and hepatorenal syndrome-AKI (HRS-AKI) in cirrhosis. ACLF, acute-on-chronic liver failure; LT, liver transplantation; RRT, renal replacement therapy; sCr, serum creatinine.

Next to the updated definition of AKI, the HRS subclassification was also revised by the ICA (2015)^16^: HRS type 1 was renamed HRS-AKI, and both the time interval for kidney function deterioration (2 weeks) and the sCr cutoff (≥2.5 mg/dL) were abandoned. Kidney dysfunction criteria for HRS-AKI (that is, dynamics of sCr levels) were aligned with general AKI criteria: sCr increase of at least 0.3 mg/dL within 48 hours or sCr increase by at least 1.5-fold from baseline (sCr levels within 3 months eligible) to reach a final sCr of at least 1.5 mg/dL (corresponding to AKI 1b). Importantly, HRS diagnosis is contingent only upon lack of improvement in kidney function (that is, decrease in sCr) within 48 hours after cessation of diuretics and volume expansion with albumin (1 g/kg per day up to a maximum of 100 g per day)^18^ (Table 1; Figure 1 and Figure 2). The revised definition of kidney dysfunction meant to facilitate an earlier diagnosis of HRS and thus reduce the time lag between worsening of kidney function and treatment initiation. This change was triggered by studies indicating that HRS reversal upon treatment with albumin and vasopressors was less likely in patients with higher sCr levels at treatment initiation, which may be indicative of a longer duration of renal impairment^21,22^.

**Table 1.**
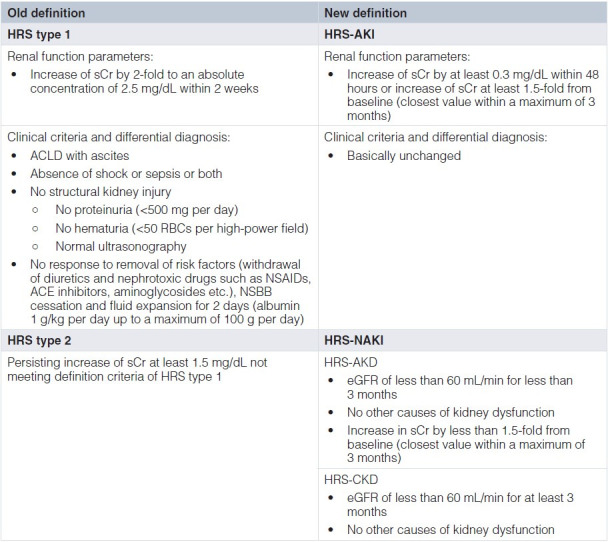
Old vs. new diagnostic criteria for hepatorenal syndrome in cirrhosis according to the International Club of Ascites and the European Association for the Study of the Liver. ACLD, advanced chronic liver disease; AKD, acute kidney disease; AKI, acute kidney injury; CKD, chronic kidney disease; eGFR, estimated glomerular filtration rate; HRS, hepatorenal syndrome; NAKI, non-acute kidney injury; NSAID, non-steroidal anti-inflammatory drug; NSBB, non-selective beta blocker; RBC, red blood cell; sCr, serum creatinine.

Moreover, HRS type 2 was renamed HRS-NAKI (that is, non-AKI), a term endorsed by the EASL (2018) on the basis of the clinical experience that the traditional HRS type 2 comprised very heterogeneous entities or courses of kidney dysfunction^23^. First, HRS-NAKI is defined by not meeting HRS-AKI criteria regarding the time course of sCr dynamics delineated above. Second, HRS-NAKI was further determined and subclassified by estimated GFR (eGFR) and the duration of kidney dysfunction on the basis of KDIGO criteria for CKD (2005)^24^: (a) an sCr increase of more than 50% or an eGFR of less than 60 mL/min per 1.73 m^2^ for less than 3 months was termed HRS-acute kidney disease (HRS-AKD), and (b) eGFR of less than 60 mL/min per 1.73 m^2^ for more than 3 months was defined as HRS-chronic kidney disease (HRS-CKD)^23^. Clinically, HRS-NAKI is typically associated with refractory ascites that is accompanied by gradual decreases in renal function; in contrast, the main clinical feature of HRS-AKI is acute renal failure.

### Definition of treatment response

Criteria for response to AKI treatment were stated within the latest ICA consensus as well as EASL clinical practice guidelines for decompensated cirrhosis and are widely accepted regardless of treatment regimens and presence of HRS: (a) “no response” is defined by the lack of AKI stage regression, (b) “partial response” is achieved by AKI stage regression, although sCr levels remain at least 0.3 mg/dL above baseline value, and (c) “full response” is determined by a return to sCr levels of less than 0.3 mg/dL above the baseline^16,23^.

## Pathophysiology of hepatorenal syndrome

### Circulatory dysfunction

The role of circulatory dysfunction in development of HRS was proposed more than 30 years ago on the basis of the consideration that increased intrahepatic vascular tone promotes the release of vasodilating molecules which lead to splanchnic vasodilation (Figure 1), a pathophysiological concept that is not specific to HRS, as it also plays a major role in portal hypertension in general^25^. Consequently, it was hypothesized that peripheral arterial vasodilation promotes kidney dysfunction that cannot be counterbalanced by activation of the renin–angiotensin–aldosterone system (RAAS) and other compensatory mechanisms^26^. This assumption was supported by a study assessing systemic and hepatic hemodynamics in patients with ascites before and after HRS development, which found that patients with HRS exhibited increased heart rate but paradoxically decreased cardiac output (possibly indicative of cirrhotic cardiomyopathy^27^) as well as lower blood pressure while the hepatic venous pressure gradient (HVPG) was higher. Moreover, the RAAS and sympathetic nervous system were highly activated^28^. Glomerular pressure may be maintained to a certain point (for example, by release of prostaglandins into afferent arterioles); however, progression of ACLD and aggravation of hyperdynamic circulation or precipitating events such as infections (or both) aggravate systemic vasodilatation and may trigger (HRS-)AKI^29^.

### Systemic inflammation

A considerable body of evidence supporting the impact of systemic inflammation (SI) on disease progression and development of extrahepatic organ—in particular, kidney dysfunction—in ACLD has accumulated in recent years. From a clinical perspective, two recent studies demonstrated the negative impact of SI on the course of ACLD in both stable^30^ and acutely decompensated^31^ patients. Similarly, SI-related kidney dysfunction is indicated by the high prevalence of systemic inflammatory response syndrome (SIRS) reported in ACLD patients with AKI^32,33^. Bacterial translocation from the gut is believed to promote SI in patients with ACLD on the basis of the pathophysiological concept that (even in the absence of evident bacterial infections) increased exposure to pathogen-/danger-associated molecular patterns stimulates inflammatory responses within the liver and other organs (Figure 1)^34^.

Accordingly, intestinal decontamination with norfloxacin decreased the expression of pro-inflammatory genes (for example, Toll-like receptor 4 [TLR4] and caspase 3) in the kidneys of rats with cirrhosis^35^, whereas ACLD patients with SI-associated kidney injury exhibited increased renal TLR4 and caspase 3 gene expression, as well as urinary TLR4 secretion, as compared with patients without kidney dysfunction^36^. Recently, cytokine profiles were assessed in patients with acute decompensation (AD) who had no renal dysfunction, hypovolemia-associated (that is, pre-renal) AKI, or HRS-AKI. The last group was characterized by significantly increased levels of different pro-inflammatory cytokines, such as interleukin-6 (IL-6), IL-8, tumor necrosis factor-alpha (TNF-α), vascular cell adhesion protein 1 (VCAM-1), fractalkine, and macrophage inflammatory protein-1 alpha (MIP-1α)^37^. Thus, an increasing number of studies point to the important direct impact of SI, which is also a central determinant of acute-on-chronic liver failure (ACLF)^31,38,39^, on HRS development.

### Cholemic nephropathy

The concept of cholemic nephropathy (CN) is based primarily on experimental studies indicating that bile acids exert toxic effects on renal tubular cells and the observation that renal tubuli are obstructed by bile casts^29^. Bile duct–ligated rats (that is, an animal model for cholestasis or cholestatic liver disease) exhibited tubular epithelial injury induced by toxic bile acids, which was ameliorated by treatment with norursodesoxycholic acid (NorUDCA)^40^. Importantly, further experimental evidence indicates that the efficacy of NorUDCA relates to the relative increase of hydrophilic bile acids, which may have properties that are less nephrotoxic^41^. Interestingly, clinical studies have linked increased serum bilirubin with non-response to albumin/terlipressin^42,43^. Accordingly, it has been speculated that hyperbilirubinemia might be indicative of an increased renal exposure to bile acids, thereby limiting the efficacy of conventional HRS therapies^42,43^. The clinical relevance of CN was further supported by a clinicopathological study in patients with extensive liver dysfunction and jaundice, reporting bile casts in 11 of 13 patients with a diagnosis of HRS^44^. That study also found a correlation between the presence of bile casts and higher serum bilirubin levels in the overall cohort^44^. However, that study neither provided reliable information on whether HRS was appropriately diagnosed in all patients (probably due to the retrospective study design) nor on HRS subtype^44^. Another study assessed kidney biopsies in patients with liver disease and either AKI or CKD and revealed that CN was exclusively found in patients with AKI, again being associated with serum and urinary bilirubin levels. Furthermore, the study suggested that loss of aquaporin 2 in collecting ducts was related to CN (which is in line with experimental data^40^) and high bilirubin levels^45^. Nevertheless, as studies in humans were observational and performed in relatively small, heterogeneous, and highly selected patient populations because of considerable safety concerns related to kidney biopsy^45^, many open questions remain. For example, it is not clear whether CN is causally linked to either incidence or reversibility of HRS (or both).

### Other pathophysiological mechanisms discussed in the context of hepatorenal syndrome

Relative adrenal insufficiency (RAI) was reported in up to 49% of patients with AD and ascites and seems to be linked to the incidence of infections (or sepsis), AKI-HRS, and mortality^46–48^. It has been hypothesized that this state is driven by constant bacterial translocation and SI, finally resulting in an inadequate synthesis of glucocorticoids^29^. Furthermore, Acevedo *et al*. demonstrated that patients with RAI exhibited lower mean arterial pressure (MAP), a trend of higher blood urea nitrogen at baseline, and increased risk of HRS type 1 (that is, HRS-AKI)^46^. In contrast, a recent study reported a similar MAP^48^. Both studies showed no significant differences in inflammatory cytokines between patients with RAI and those without RAI^46,48^. Of note, sCr levels at the time of the adrenocorticotropic hormone stimulation test were not significantly different in any of the studies mentioned above^46–48^. Thus, the available evidence is not sufficient to draw firm conclusions on whether RAI promotes or aggravates HRS and whether glucocorticoid therapy may exert positive effects in this setting.

Furthermore, intra-abdominal hypertension (IAH) is believed to impact on HRS or AKI (or both) in patients with ascites. Previous studies in patients with tense ascites and HRS demonstrated that large-volume paracentesis (LVP) ameliorated IAH, which was paralleled by increases of creatinine clearance and cardiac output as well as decreases of systemic vascular resistance and renal resistive index^49–51^; this was possibly due to a reduction of RAAS activation^51^. It has been suggested that bedside echocardiography may be useful for assessing volume status and the presence of IAH by measurement of inferior vena cava diameter (IVCD) and inferior vena cava collapsibility index (IVCCI): Those with an IVCD of less than 1.3 cm and IVCCI of more than 40% were reclassified as fluid-depleted, those with an IVCD of more than 2 cm and IVCCI of less than 40% were considered fluid-overloaded, while those with an IVCD of less than 1.3 cm and IVCCI of less than 40% were classified as having IAH. Accordingly, this assessment could guide therapeutic decisions on fluid therapy and the need for an additional LVP^52^.

## Differential diagnosis and novel biomarkers

Current diagnostic criteria for HRS include the absence of structural kidney injury, meaning that risk factors for (for example, nephrotoxic medication and septic shock) or evidence of kidney damage (that is, proteinuria of more than 500 mg per day, hematuria of more than 50 red blood cells per high-power field, or abnormal renal ultrasound) preclude the diagnosis of HRS-AKI. Of note, the paradigm of the absence of structural damage in patients with HRS has never been proven by biopsy-controlled studies. However, early studies indicated that HRS is caused primarily by functional renal failure: A study published in 1969 reported that kidneys from patients dying from “HRS” were successfully transplanted (that is, improvement of renal function) in 6 of 7 organ recipients and achieved stable improvement (≥6 months) of renal function in 4 of 7 patients^53^. Similarly, a publication from 1970 showed that impaired renal perfusion in cirrhotic patients with kidney dysfunction was caused by vasoconstriction, which disappeared post-mortem^54^. Of note, these studies were conducted before current diagnostic criteria of HRS were established. Nevertheless, more recent studies revealed that most patients with HRS exhibit sustainable improvement of renal function after liver transplantation (LT), which supports the concept of functional kidney impairment in HRS^55,56^.

Given the complex pathophysiology of HRS-AKI, nailing it down to one main pathogenetic factor is often challenging in clinical practice, where things are usually not just black or white but rather shades of grey; this unveils the difficulty of finding the most accurate diagnosis or treatment strategy^18^. Various types of AKI—pre-renal AKI, post-renal AKI, HRS-AKI, and acute tubular necrosis (ATN-)AKI—should be considered in patients with ACLD (Figure 1). Prerenal AKI should respond to the cessation of diuretic treatment, fluid replacement, and albumin treatment, while postrenal AKI—though comparatively rare—should always be excluded by ultrasound. However, HRS-AKI cannot be reliably differentiated from ATN-AKI (that is, structural kidney damage) by simple tests. Fractional urinary sodium excretion (FeNa) was considered for differential diagnosis, supported by data indicating the good discriminatory value of FeNa for ATN^57^. However, FeNa displayed a poor predictive value for HRS diagnosis in a study of LT candidates with renal dysfunction undergoing kidney biopsy and even patients with biopsy-proven ATN exhibited low FeNa^58^. Thus, because of concerns towards the impact of diuretic use on sodium excretion, FeNa has not been implemented in the HRS-AKI diagnostic criteria^29^. Urinary biomarkers of tubular injury emerged in recent years. For example, Belcher *et al*. reported increased levels of kidney injury molecule-1 (KIM 1), IL-18, liver-type fatty acid binding protein (L-FABP), albumin, and neutrophil gelatinase-associated lipocalin (NGAL) in patients with ATN-AKI^59^. NGAL performed particularly well for differentiating between ATN and other types of AKI (area-under-the-receiver operating characteristics [AUROCs] of at least 0.80)^60,61^. Interestingly, in a subsequent study by Huelin *et al*., NGAL displayed an even better diagnostic performance when assessed on day 3 (AUROC 0.87 vs. 0.80 on day 1)^61^. Nevertheless, about 10% of patients with prerenal AKI and HRS-AKI displayed NGAL levels above the proposed cutoff for identification of ATN, which may indicate that tubular damage occurs in these clinical entities as well^61^. Unfortunately, these and other studies were based only on clinical and laboratory discrimination for differential diagnosis of AKI (particularly relevant for ATN vs. HRS) while renal biopsies as the diagnostic gold standard were not performed. In this regard, the limited diagnostic performance of these markers could also be explained by the inherent limitations of the imperfect reference standards applied in these studies.

## Management 

### Removal of risk factors and fluid challenge

Because the latest definition criteria of HRS abandoned the mandatory requirement of an absolute sCr cutoff at 2.5 mg/dL, early diagnosis of and treatment uptake for (HRS-)AKI are now facilitated; this may lead to improved (HRS-)AKI reversal given that HRS reversal by vasoconstrictor treatment was contingent on sCr values at treatment initiation in previous studies^21,22^. The initial measures after diagnosis of AKI include removal of risk factors for kidney dysfunction, namely withdrawal of nephrotoxic drugs (for example, non-steroidal anti-inflammatory drugs and angiotensin-converting enzyme inhibitors) and diuretics, treatment of infections, and fluid therapy depending on volume status (see above) and AKI grade (Table 1; Figure 2)^29^. Importantly, this also includes the (at least contemporary) cessation of non-selective beta blocker (NSBB) treatment, as previous studies have reported an increased risk of AKI or HRS during circulatory stress in patients receiving NSBB therapy^62^, which is most likely related to its impact on cardiac output^63^. However, if the benefits still outweigh the potential risks in the individual patient, it seems that NSBB treatment can be safely reinitiated when AKI is resolved^64,65^.

### Albumin

Besides removal of risk factors, AKI grade will determine the necessity and time point of plasma volume expansion with albumin. Current EASL clinical practice guidelines recommend that patients presenting with AKI 1b or higher receive albumin for two consecutive days (1 g/kg body weight up to a maximum of 100 g per day) (Figure 2). Conversely, patients with AKI 1a should be monitored for 48 hours after removal of risk factors, followed by the same albumin protocol if kidney dysfunction progresses to AKI 1b or higher; if AKI 1a persists, the treatment decision should be individualized^23^. The differentiation between AKI 1a and 1b is supported by the observation that AKI 1a is caused by prerenal AKI in about half of patients (vs. 25–30% in AKI 1b), resolves in about 90% of patients (vs. about 50% in AKI 1b), and only rarely progresses to HRS-AKI (14% vs. 34% in AKI 1b)^66^.

In recent decades, evidence of the beneficial effects of albumin has accumulated in certain settings of ACLD. Albumin is currently indicated for the prevention of circulatory dysfunction after LVP, HRS prevention in patients with spontaneous bacterial peritonitis (SBP), and HRS diagnosis as well as therapy in combination with vasoconstrictors^23^. The treatment rationale of albumin use was initially based on its beneficial hemodynamic effect on circulatory dysfunction because of its properties as a plasma expander; however, more recent studies also indicate that albumin modulates SI by binding and thus scavenging or inactivating pro-inflammatory molecules^67–70^. Furthermore, albumin improves the autoregulation of renal perfusion which may be attributed to a reduction of SI, oxidative stress, and endothelial activation^71^.

The beneficial effect of albumin in the prevention of post-LVP circulatory dysfunction was established more than two decades ago, as studies demonstrated that renal function and RAAS activation were ameliorated (or at least stabilized) by albumin infusion^72–74^. Of note, compared with other plasma expanders, albumin exhibited superior efficiency, indicating that additional biological properties of albumin may contribute to its therapeutic effects^75^. Similarly, an RCT showed that patients with SBP were less likely to develop renal impairment when receiving albumin on top of antibiotic therapy^76^, which was further supported by the randomized comparison between albumin and hydroxyethyl starch in patients with SBP, which also found that albumin improved markers of endothelial dysfunction and SI^77^. Importantly, prior studies in patients with HRS also displayed improved renal function upon albumin treatment^51^ and found that the use of albumin on top of vasoconstrictors improves the chances of HRS reversal^78^.

Recently, several RCTs have evaluated the effects of pre-emptive long-term albumin administration. The ANSWER study (not placebo-controlled) has investigated the effects of weekly albumin infusion (40 g twice weekly for 2 weeks followed by 40 g per week) for 18 months in patients with uncomplicated ascites and revealed that patients receiving albumin exhibited better ascites control (hazard ratio [HR] of 0.48 for the incidence of paracentesis, HR of 0.43 for developing refractory ascites), a decreased risk of renal dysfunction (HR of 0.50), and particularly HRS-AKI incidence (HR of 0.39). Finally, these effects translated into reduced mortality (HR of 0.62)^79^. A subsequent observational study in patients with refractory ascites found reduced mortality and hospitalization and a trend of a reduced HRS incidence^80^. Moreover, recently published data from two RCTs on short-term (INFECIR-2 study of patients with non-SBP bacterial infections) and long-term (pilot PRECIOSA study of patients without infection) albumin treatment exhibited reductions of SI and circulatory dysfunction in patients with decompensated ACLD. Of note, in the pilot PRECIOSA study on long-term albumin therapy, this effect was restricted to the high-dose (1.5 g/kg body weight per week) group. Nevertheless, whereas these trials provided evidence for the long-term use of albumin in patients with ascites (that is, patients at risk for kidney dysfunction and HRS), another RCT comprising a placebo group reported a lack of efficacy of long-term low-dose (40 g/15 days) albumin therapy in more advanced patients: Albumin in combination with midodrine (an alpha-1 adrenergic agonist) failed to prevent complications (including renal dysfunction) in patients awaiting LT^81^. The PRECIOSA study (NCT03451292; 1.5 g/kg body weight per week after hospital discharge in patients with uncomplicated ascites) will provide further insights into the efficacy of different pre-emptive albumin administration regimens.

In contrast, the results from the ATTIRE RCT (14 days of albumin treatment to achieve serum albumin levels of at least 35 g/L vs. standard care in patients with AD and serum albumin levels of less than 30 g/L) were recently published and indicated no beneficial effect of albumin on the primary endpoint (composite of infection, renal dysfunction, or mortality between days 3 and 15 after treatment)^82^. Furthermore, the INFECIR-1/-2 an ALB-CIRINF trials (investigating the use of albumin in patients with ACLD and non-SBP bacterial infections) found no significant impact on kidney dysfunction^83–85^. Moreover, the ALB-CIRINF study and the ATTIRE trial reported increased occurrence of pulmonary edema (putatively associated with volume overload) in the albumin groups, indicating that the indiscriminate short-term use of albumin not only may lack therapeutic benefit but also may lead to adverse events^82,83^.

Because these studies are (or were) conducted in distinct patient populations and provided divergent results, the identification of those who benefit most from pre-emptive albumin treatment is crucial for treatment individualization and optimization of resource allocation. Because the therapeutic effect of albumin seemed to differ between the ANSWER and the ATTIRE studies, which addressed different patient groups, pre-emptive albumin treatment may be effective only within a certain therapeutic window and in distinct clinical settings. Furthermore, high doses of albumin seemed to be required to obtain a clinical benefit.

### Vasoactive drugs

Vasoactive drugs are the other cornerstone of HRS therapy, as these pharmacological agents reduce splanchnic pooling and increase the effectively circulating blood volume and thus may improve renal perfusion (next to plasma expansion by albumin). Multiple studies, including RCTs, have confirmed the efficacy of vasoconstrictors for HRS-AKI therapy^86,87^. Terlipressin (which has yet to be approved in the US) is the first-line treatment recommended by EASL; however, noradrenaline, as well as midodrine in combination with octreotide, has also shown some efficacy for HRS therapy^23,29^.

Although all these drugs are vasoconstrictors, they have distinct properties that might relate to differences in efficacy: Terlipressin is a vasopressin analogue and exerts systemic and splanchnic vasoconstriction via vasopressin 1A receptor activation, while agonism at vasopressin 1B receptors activates the adrenocorticotropic hormone–cortisol axis which may be relevant for HRS because of the amelioration of RAI^88,89^. Accordingly, terlipressin decreases HVPG while raising MAP. Noradrenaline and midodrine increase the vascular tone and thus MAP (and possibly HVPG) by activating α1-adrenergic receptors. However, whereas noradrenalin and terlipressin are potent vasopressors, midodrine achieves only modest increases in MAP. Octreotide is a somatostatin analogue and thus promotes splanchnic vasoconstriction^29^, thereby decreasing HVPG.

Of note, terlipressin may be given by intravenous boluses, but owing to its stability at room temperature^90^ as well as the lower required dose and a favorable side effect profile^91^, continuous infusion (2 mg per day up to a maximum of 12 mg per day, via central or peripheral venous catheter) should be preferred^92^. Of note, the recommended initial dosing regimen of continuous terlipressin infusion^23^ is based mostly on the study design of the trial by Cavallin *et al*.^91^ and may need further refinement in the future. Noradrenaline is administered by continuous infusion and requires a central venous catheter. Octreotide is usually administered subcutaneously every 8 hours or as a continuous infusion, whereas midodrine is given orally.

Several studies have indicated that the efficacy of midodrine in combination with octreotide for HRS therapy is inferior to that of other vasoconstrictors^87,93,94^. Therefore, this review will focus on relevant advances regarding studies on terlipressin and noradrenaline.

Multiple (non)randomized and (non)controlled trials have demonstrated the efficacy of terlipressin on HRS during the last two decades^21,78,91,93,95–98^. For HRS-AKI, noradrenaline has exhibited an efficacy similar to that of terlipressin in several (non)randomized and (non)controlled trials; however, available trials were likely underpowered to confidently establish the non-inferiority (or possibly even superiority) of noradrenaline^99–101^. Efforts to combine available trials in meta-analyses were unable to provide clear evidence in the comparison between terlipressin and noradrenalin (especially evidence of a survival benefit) and this was due primarily to trial design differences that limited the interpretability of comparisons^87,94,102^. Importantly, predictors of non-response to HRS therapy include sCr levels^21,22^, high model for end-stage liver disease (MELD) score, sepsis^98^, and ACLF severity^103^. Thus, differences in patient selection directly impact on the efficacy of HRS therapy, which further limits between-study comparisons. Therefore, the harmonization of study design and proper sample size seems crucial for future trials.

Recent RCTs have confirmed the dilemma that different patient populations and HRS definitions may complicate interpretability of study results: The REVERSE trial did not detect a significant benefit by terlipressin on HRS reversal (defined by two sCr values below 1.5 mg/dL within 48 hours)^97^. Of note, sCr significantly decreased in the terlipressin group; in general, achieving an sCr decrease was associated with improved outcomes. HRS-AKI was defined by the old ICA criteria (that is, doubling of sCr to at least 2.5 mg/dL within 2 weeks^20^) in that study^97^. However, one post-hoc analysis and one meta-analysis considering patients from this trial reported that patients receiving terlipressin had higher chances of HRS reversal^104,105^. More specifically, the post-hoc analysis by Wong *et al*. reported that patients with SIRS were significantly more likely to achieve HRS reversal when they received terlipressin (32% vs. 3% receiving placebo)^104^. The results of the CONFIRM trial (RCT comparing terlipressin vs. placebo) comprising a considerably higher number of patients, as compared to previous studies, were recently published and clearly established the efficacy of terlipressin on reversal of HRS type 1 (32% vs. 17% in placebo group). Of note, the old ICA criteria^20^ (sCr cut-off of 2.25 instead of of 2.5 mg/dL) were applied in that study, probably delaying treatment initiation^106^. However, that study found no difference in overall and transplant-free survival (about 50% in both groups) after 90 days, underlining the paramount importance of liver transplantation to improve outcomes beyond kidney function. Respiratory failure tended to be more common in the terlipressin arm, as did death due to respiratory failure, which may be explained by the development of pulmonary edema due to increases in afterload. Importantly, the risk of this potentially lethal complication may be reduced by continuous infusion^91^.

Finally, a recent RCT by Arora *et al*. directly compared terlipressin with noradrenaline in patients with HRS-AKI and ACLF^107^. Terlipressin exhibited superior efficacy over noradrenaline in HRS reversal (40% vs. 17% in the noradrenaline group), reduced the need for RRT (57% vs. 80%), and ameliorated prognosis (28-day survival: 48% vs. 20%). Importantly, that study used most recent AKI staging and treatment response criteria of the ICA and EASL^107^ and may represent one of the first RCTs with an adequate sample size to support the rationale for abandoning absolute sCr values and 2 weeks latency for HRS diagnosis (that is, improved treatment success by early treatment initiation). Moreover, it seems that terlipressin is particularly effective in the context of SIRS/ACLF and should be preferred over noradrenaline in this setting.

### Antibiotics

Given the pathophysiological concept of the impact of SI on kidney dysfunction in ACLD and the close association between AKI and SBP^108^, antibiotic prophylaxis may display beneficial effects on HRS. Consequently, an RCT by Fernández *et al*. showed that in selected patients with ascites at high risk for SBP, primary prophylaxis with norfloxacin reduced the risk of HRS (28% vs. 41% in the placebo group)^109^. The protective effect on HRS development was not confirmed by the NORFLOCIR study (RCT norfloxacin vs. placebo in patients with Child–Pugh stage C)^110^. However, the results of that study should be interpreted with caution, as it was not powered to evaluate this endpoint and included a high proportion of patients with severe alcoholic hepatitis. Moreover, a broadening of the prophylactic use of antibiotics is controversial because of the potential emergence of antibiotic resistance, although the global study initiated by the ICA did not observe an association between prophylactic quinolone use and infections with multidrug-resistant bacteria^111^. Finally, results of the LIVERHOPE efficacy trial (NCT03780673; RCT on rifaximin and simvastatin vs. placebo) are awaited, as they may provide further insights into the efficacy of poorly adsorbable antibiotics for preventing HRS development in patients with decompensated ACLD.

### Transjugular intrahepatic portosystemic shunt

Patients with ascites are at risk of developing SBP or HRS-(N)AKI or both; thus, interventions targeting incidence of ascites or ascites control will likely ameliorate these downstream complications^112^. Given that minor reductions of portal pressure by medical therapies translate into reduced risks of developing refractory ascites and HRS^113,114^, transjugular intrahepatic portosystemic shunt (TIPS) implantation may prevent the development of HRS-(N)AKI^112^. TIPS placement exerted beneficial effects on RAAS and sympathetic activity^115^ and significantly reduced HRS incidence in patients with refractory ascites (9% vs. 31% receiving LVP and albumin)^116^ and thus is an interesting treatment option to prevent and treat HRS-NAKI (that is, former type 2), which typically is associated with recurrent or refractory ascites^117^. In this regard, a comparison of the results of the most recent RCT^118^ with earlier studies indicates that TIPS may be particularly effective (in terms of preventing mortality) if placed in patients with recurrent (that is, tense ascites that recurred on at least three occasions within a 12-month period despite therapeutic measures) rather than refractory ascites as defined by ICA criteria^112^. Moreover, a small observational study reported that patients with HRS-AKI (that is, former type 1) receiving TIPS displayed increased GFR and urinary sodium excretion^119^. Nevertheless, further and larger trials are warranted to consolidate the efficacy of covered TIPS grafts for prevention and treatment of renal dysfunction and to weigh these potential beneficial effects against adverse events such as hepatic encephalopathy. The use of small-diameter or underdilated covered controlled expansion stents may prove useful in this regard^112,120,121^, as recent non-randomized studies showed a reduced incidence of hepatic encephalopathy and similar efficacy for ascites control and prevention of variceal bleeding^122,123^.

### Renal replacement therapy, liver transplantation, and combined liver-kidney transplantation

RRT is considered merely a bridging treatment to LT^124^ on the basis of the observation that RRT for HRS-AKI does not improve the prognosis^125^, and patients with renal failure and/or RRT who were either not listed or not transplanted exhibited exceedingly high (85–95%) short- and mid-term mortality rates^126–128^. A large study comprising more than 2,000 patients found that only 9% of patients on RRT before LT developed a need for chronic dialysis within 6 months^55^. Importantly, Piano *et al*. recently reported that LT waitlist patients with HRS-AKI responding to terlipressin and albumin exhibited a lower risk of requiring RRT and developing CKD after LT^129^. In contrast, patients with HRS-NAKI responding to terlipressin (61%) displayed high relapse rates after cessation of treatment (pre-LT; 58%) and no significantly improved kidney outcomes post-LT^130^. Again, these data underline the clinical diversity between HRS-AKI und HRS-NAKI and point to early and consequent identification of HRS subtype and the importance of individualized treatment strategies.

Since both HRS-AKI and HRS-NAKI mostly resolve after LT, simultaneous liver and kidney transplantation (SLKT) remains disputed in the context of HRS^23^. Previous studies have identified risk factors for persistent renal dysfunction after LT, including the need for and duration of RRT and time period of AKI before LT as well as age and diabetes^55,131^. According to current recommendations, SLKT should be considered if patients (a) have AKI stage 3, (b) exhibit an eGFR of not more than 35 mL/min or GFR of not more than 25 mL/min (that is, iothalamate clearance), or (c) require RRT or a combination of these factors. More specifically, these three scenarios (which may occur alone or in combination) should last for more than 4 weeks, counted from the onset of any of these criteria^23,132^. Sequential transplantation of liver and kidney (as an alternative for SLKT) in patients with HRS was discussed as a potential strategy to avoid futile kidney transplantations in patients who would recover from LT alone and thus optimize organ utilization^133^. However, this proposal is based primarily on retrospective studies that did not systematically address (or even exclude^134^) patients with HRS; this underlines that more reliable studies on patient selection are warranted.

## Conclusions

Recent changes in the definition and diagnosis of HRS have facilitated early recognition and treatment initiation, which may result in better chances of reversal and survival of HRS-AKI. Albumin in combination with vasoconstrictor therapy remains the medical cornerstone therapy of HRS-AKI. Recent data suggest that terlipressin should be preferred over noradrenaline for treatment of HRS-AKI in the context of ACLF. Nevertheless, mortality of patients with HRS remains high, indicating that prevention of HRS and associated pathomechanisms—such as bacterial translocation and SI—should be the focus of further research. To this end, development of pathophysiology-related biomarkers and disease-modifying HRS treatments targeting SI or hyperdynamic circulation (or both) are urgently needed.

## Abbreviations

ACLD, advanced chronic liver disease; ACLF, acute-on-chronic liver failure; AD, acute decompensation; AKI, acute kidney injury; AKIN, Acute Kidney Injury Network; ATN, acute tubular necrosis; AUROC, area-under-the-receiver operating characteristic; CKD, chronic kidney disease; CN, cholemic nephropathy; EASL, European Association for the Study of the Liver; eGFR, estimated glomerular filtration rate; FeNa, fractional urinary sodium excretion; GFR, glomerular filtration rate; HR, hazard ratio; HRS, hepatorenal syndrome; HVPG, hepatic venous pressure gradient; IAH, intra-abdominal hypertension; ICA, International Club of Ascites; IL, interleukin; IVCCI, inferior vena cava collapsibility index; IVCD, inferior vena cava diameter; KDIGO, Kidney Disease: Improving Global Outcomes; LT, liver transplantation; LVP, large-volume paracentesis; MAP, mean arterial pressure; NAKI, non-acute kidney injury; NGAL, neutrophil gelatinase-associated lipocalin; NorUDCA, norursodesoxycholic acid; NSBB, non-selective beta blocker; RAAS, renin–angiotensin–aldosterone system; RAI, relative adrenal insufficiency; RCT, randomized controlled trial; RRT, renal replacement therapy; SBP, spontaneous bacterial peritonitis; sCr, serum creatinine; SI, systemic inflammation; SIRS, systemic inflammatory response syndrome; SLKT, simultaneous liver and kidney transplantation; TIPS, transjugular intrahepatic portosystemic shunt; TLR4, Toll-like receptor 4

## References

[1] Sherman DS Fish DN Teitelbaum I: Assessing renal function in cirrhotic patients: Problems and pitfalls. *Am J Kidney Dis.* 2003; 41(2): 269–78. 10.1053/ajkd.2003.50035 12552488

[2] Bellomo R Ronco C Kellum JA: Acute renal failure - definition, outcome measures, animal models, fluid therapy and information technology needs: The Second International Consensus Conference of the Acute Dialysis Quality Initiative (ADQI) Group. *Crit Care.* 2004; 8(4): R204–12. 10.1186/cc2872 15312219 PMC522841

[3] Mehta RL Kellum JA Shah SV: Acute Kidney Injury Network: Report of an initiative to improve outcomes in acute kidney injury. *Crit Care.* 2007; 11(2): R31. 10.1186/cc5713 17331245 PMC2206446

[4] Chertow GM Burdick E Honour M: Acute kidney injury, mortality, length of stay, and costs in hospitalized patients. *J Am Soc Nephrol.* 2005; 16(11): 3365–70. 10.1681/ASN.2004090740 16177006

[5] Lassnigg A Schmidlin D Mouhieddine M: Minimal changes of serum creatinine predict prognosis in patients after cardiothoracic surgery: A prospective cohort study. *J Am Soc Nephrol.* 2004; 15(6): 1597–605. 10.1097/01.asn.0000130340.93930.dd 15153571

[6] Levy MM Macias WL Vincent JL: Early changes in organ function predict eventual survival in severe sepsis. *Crit Care Med.* 2005; 33(10): 2194–201. 10.1097/01.ccm.0000182798.39709.84 16215369

[7] Summary of Recommendation Statements. *Kidney Int Suppl (2011).* 2012; 2(1): 8–12. 10.1038/kisup.2012.7 25018916 PMC4089654

[8] Arroyo V Ginès P Gerbes AL: Definition and diagnostic criteria of refractory ascites and hepatorenal syndrome in cirrhosis. International Ascites Club. *Hepatology.* 1996; 23(1): 164–76. 10.1002/hep.510230122 8550036

[9] Piano S Rosi S Maresio G: Evaluation of the Acute Kidney Injury Network criteria in hospitalized patients with cirrhosis and ascites. *J Hepatol.* 2013; 59(3): 482–9. 10.1016/j.jhep.2013.03.039 23665185

[10] Belcher JM Garcia-Tsao G Sanyal AJ: Association of AKI with mortality and complications in hospitalized patients with cirrhosis. *Hepatology.* 2013; 57(2): 753–62. 10.1002/hep.25735 22454364 PMC3390443

[11] Tsien CD Rabie R Wong F: Acute kidney injury in decompensated cirrhosis. *Gut.* 2013; 62(1): 131–7. 10.1136/gutjnl-2011-301255 22637695

[12] de Carvalho JR Villela-Nogueira CA Luiz RR: Acute kidney injury network criteria as a predictor of hospital mortality in cirrhotic patients with ascites. *J Clin Gastroenterol.* 2012; 46(3): e21–6. 10.1097/MCG.0b013e31822e8e12 21934526

[13] Wong F O’Leary JG Reddy KR: New consensus definition of acute kidney injury accurately predicts 30-day mortality in patients with cirrhosis and infection. *Gastroenterology.* 2013; 145(6): 1280–8.e1. 10.1053/j.gastro.2013.08.051 23999172 PMC4418483

[14] Altamirano J Fagundes C Dominguez M: Acute kidney injury is an early predictor of mortality for patients with alcoholic hepatitis. *Clin Gastroenterol Hepatol.* 2012; 10(1): 65–71.e3. 10.1016/j.cgh.2011.09.011 21946124

[15] Fagundes C Barreto R Guevara M: A modified acute kidney injury classification for diagnosis and risk stratification of impairment of kidney function in cirrhosis. *J Hepatol.* 2013; 59(3): 474–81. 10.1016/j.jhep.2013.04.036 23669284

[16] Angeli P Gines P Wong F: Diagnosis and management of acute kidney injury in patients with cirrhosis: Revised consensus recommendations of the International Club of Ascites. *Gut.* 2015; 64(4): 531–7. 10.1136/gutjnl-2014-308874 25631669

[17] Angeli P Gatta A Caregaro L: Tubular site of renal sodium retention in ascitic liver cirrhosis evaluated by lithium clearance. *Eur J Clin Invest.* 1990; 20(1): 111–7. 10.1111/j.1365-2362.1990.tb01800.x 2108033

[18] Angeli P Garcia-Tsao G Nadim MK: News in pathophysiology, definition and classification of hepatorenal syndrome: A step beyond the International Club of Ascites (ICA) consensus document. *J Hepatol.* 2019; 71(4): 811–22. 10.1016/j.jhep.2019.07.002 31302175

[19] Amathieu R Al-Khafaji A Sileanu FE: Significance of oliguria in critically ill patients with chronic liver disease. *Hepatology.* 2017; 66(5): 1592–600. 10.1002/hep.29303 28586126

[20] Salerno F Gerbes A Ginès P: Diagnosis, prevention and treatment of hepatorenal syndrome in cirrhosis. *Postgrad Med J.* 2008; 84(998): 662–70. 10.1136/gut.2006.107789 19201943

[21] Martín-Llahí M Pépin MN Guevara M: Terlipressin and albumin vs albumin in patients with cirrhosis and hepatorenal syndrome: A randomized study. *Gastroenterology.* 2008; 134(5): 1352–9. 10.1053/j.gastro.2008.02.024 18471512

[22] Boyer TD Sanyal AJ Garcia-Tsao G: Predictors of response to terlipressin plus albumin in hepatorenal syndrome (HRS) type 1: Relationship of serum creatinine to hemodynamics. *J Hepatol.* 2011; 55(2): 315–21. 10.1016/j.jhep.2010.11.020 21167235 PMC3728672

[23] European Association for the Study of the Liver: EASL Clinical Practice Guidelines for the management of patients with decompensated cirrhosis. *J Hepatol.* 2018; 69(2): 406–60. 10.1016/j.jhep.2018.03.024 29653741

[24] Levey AS Eckardt KU Tsukamoto Y: Definition and classification of chronic kidney disease: A position statement from Kidney Disease: Improving Global Outcomes (KDIGO). *Kidney Int.* 2005; 67(6): 2089–100. 10.1111/j.1523-1755.2005.00365.x 15882252

[25] Bosch J Groszmann RJ Shah VH: Evolution in the understanding of the pathophysiological basis of portal hypertension: How changes in paradigm are leading to successful new treatments. *J Hepatol.* 2015; 62(1 Suppl): S121–30. 10.1016/j.jhep.2015.01.003 25920081 PMC4519833

[26] Schrier RW Arroyo V Bernardi M: Peripheral arterial vasodilation hypothesis: A proposal for the initiation of renal sodium and water retention in cirrhosis. *Hepatology.* 1988; 8(5): 1151–7. 10.1002/hep.1840080532 2971015

[27] Izzy M vanWagner LB Lin G: Redefining Cirrhotic Cardiomyopathy for the Modern Era. *Hepatology.* 2020; 71(1): 334–45. 10.1002/hep.30875 31342529 PMC7288530

[28] Ruiz-del-Arbol L Monescillo A Arocena C: Circulatory function and hepatorenal syndrome in cirrhosis. *Hepatology.* 2005; 42(2): 439–47. 10.1002/hep.20766 15977202

[29] Simonetto DA Gines P Kamath PS: Hepatorenal syndrome: Pathophysiology, diagnosis, and management. *BMJ.* 2020; 370: m2687. 10.1136/bmj.m2687 32928750

[30] Costa D Simbrunner B Jachs M: Systemic inflammation increases across distinct stages of advanced chronic liver disease and correlates with decompensation and mortality. *J Hepatol.* 2021; 74(4): 819–828. 10.1016/j.jhep.2020.10.004 33075344

[31] Trebicka J Fernandez J Papp M: The PREDICT study uncovers three clinical courses of acutely decompensated cirrhosis that have distinct pathophysiology. *J Hepatol.* 2020; 73(4): 842–854. 10.1016/j.jhep.2020.06.013 32673741

[32] Thabut D Massard J Gangloff A: Model for end-stage liver disease score and systemic inflammatory response are major prognostic factors in patients with cirrhosis and acute functional renal failure. *Hepatology.* 2007; 46(6): 1872–82. 10.1002/hep.21920 17972337

[33] Maiwall R Chandel SS Wani Z: SIRS at Admission Is a Predictor of AKI Development and Mortality in Hospitalized Patients with Severe Alcoholic Hepatitis. *Dig Dis Sci.* 2016; 61(3): 920–9. 10.1007/s10620-015-3921-4 26470868

[34] Simbrunner B Mandorfer M Trauner M: Gut-liver axis signaling in portal hypertension. *World J Gastroenterol.* 2019; 25(39): 5897–5917. 10.3748/wjg.v25.i39.5897 31660028 PMC6815800

[35] Shah N Dhar D El Zahraa Mohammed F: Prevention of acute kidney injury in a rodent model of cirrhosis following selective gut decontamination is associated with reduced renal TLR4 expression. *J Hepatol.* 2012; 56(5): 1047–1053. 10.1016/j.jhep.2011.11.024 22266601

[36] Shah N Mohamed FE Jover-Cobos M: Increased renal expression and urinary excretion of TLR4 in acute kidney injury associated with cirrhosis. *Liver Int.* 2013; 33(3): 398–409. 10.1111/liv.12047 23402610

[37] Solé C Solà E Huelin P: Characterization of inflammatory response in hepatorenal syndrome: Relationship with kidney outcome and survival. *Liver Int.* 2019; 39(7): 1246–1255. 10.1111/liv.14037 30597709 PMC6767546

[38] Clària J Stauber RE Coenraad MJ: Systemic inflammation in decompensated cirrhosis: Characterization and role in acute-on-chronic liver failure. *Hepatology.* 2016; 64(4): 1249–64. 10.1002/hep.28740 27483394

[39] Moreau R Jalan R Gines P: Acute-on-chronic liver failure is a distinct syndrome that develops in patients with acute decompensation of cirrhosis. *Gastroenterology.* 2013; 144(7): 1426–37, 1437.e1–9. 10.1053/j.gastro.2013.02.042 23474284

[40] Fickert P Krones E Pollheimer MJ: Bile acids trigger cholemic nephropathy in common bile-duct-ligated mice. *Hepatology.* 2013; 58(6): 2056–69. 10.1002/hep.26599 23813550

[41] Krones E Eller K Pollheimer MJ: NorUrsodeoxycholic acid ameliorates cholemic nephropathy in bile duct ligated mice. *J Hepatol.* 2017; 67(1): 110–119. 10.1016/j.jhep.2017.02.019 28242240

[42] Nazar A Pereira GH Guevara M: Predictors of response to therapy with terlipressin and albumin in patients with cirrhosis and type 1 hepatorenal syndrome. *Hepatology.* 2010; 51(1): 219–26. 10.1002/hep.23283 19877168

[43] Barreto R Fagundes C Guevara M: Type-1 hepatorenal syndrome associated with infections in cirrhosis: Natural history, outcome of kidney function, and survival. *Hepatology.* 2014; 59(4): 1505–13. 10.1002/hep.26687 24037970

[44] van Slambrouck CM Salem F Meehan SM: Bile cast nephropathy is a common pathologic finding for kidney injury associated with severe liver dysfunction. *Kidney Int.* 2013; 84(1): 192–7. 10.1038/ki.2013.78 23486516

[45] Bräsen JH Mederacke YS Schmitz J: Cholemic Nephropathy Causes Acute Kidney Injury and Is Accompanied by Loss of Aquaporin 2 in Collecting Ducts. *Hepatology.* 2019; 69(5): 2107–2119. 10.1002/hep.30499 30633816

[46] Acevedo J Fernández J Prado V: Relative adrenal insufficiency in decompensated cirrhosis: Relationship to short-term risk of severe sepsis, hepatorenal syndrome, and death. *Hepatology.* 2013; 58(5): 1757–65. 10.1002/hep.26535 23728792

[47] Jang JY Kim TY Sohn JH: Relative adrenal insufficiency in chronic liver disease: Its prevalence and effects on long-term mortality. *Aliment Pharmacol Ther.* 2014; 40(7): 819–26. 10.1111/apt.12891 25078874

[48] Piano S Favaretto E Tonon M: Including Relative Adrenal Insufficiency in Definition and Classification of Acute-on-Chronic Liver Failure. *Clin Gastroenterol Hepatol.* 2020; 18(5): 1188–1196.e3. 10.1016/j.cgh.2019.09.035 31589973

[49] Umgelter A Reindl W Franzen M: Renal resistive index and renal function before and after paracentesis in patients with hepatorenal syndrome and tense ascites. *Intensive Care Med.* 2009; 35(1): 152–6. 10.1007/s00134-008-1253-y 18802688

[50] Umgelter A Reindl W Wagner KS: Effects of plasma expansion with albumin and paracentesis on haemodynamics and kidney function in critically ill cirrhotic patients with tense ascites and hepatorenal syndrome: A prospective uncontrolled trial. *Crit Care.* 2008; 12(1): R4. 10.1186/cc6765 18197961 PMC2374626

[51] Umgelter A Wagner KS Reindl W: Renal and circulatory effects of large volume plasma expansion in patients with hepatorenal syndrome type 1. *Ann Hepatol.* 2012; 11(2): 232–9. 22345341

[52] Velez JCQ Petkovich B Karakala N: Point-of-Care Echocardiography Unveils Misclassification of Acute Kidney Injury as Hepatorenal Syndrome. *Am J Nephrol.* 2019; 50(3): 204–211. 10.1159/000501299 31394538

[53] Koppel MH Coburn JW Mims MM: Transplantation of cadaveric kidneys from patients with hepatorenal syndrome. Evidence for the functionalnature of renal failure in advanced liver disease. *N Engl J Med.* 1969; 280(25): 1367–71. 10.1056/NEJM196906192802501 4890476

[54] Epstein M Berk DP Hollenberg NK: Renal failure in the patient with cirrhosis. The role of active vasoconstriction. *Am J Med.* 1970; 49(2): 175–85. 10.1016/s0002-9343(70)80073-0 5452940

[55] Sharma P Goodrich NP Zhang M: Short-term pretransplant renal replacement therapy and renal nonrecovery after liver transplantation alone. *Clin J Am Soc Nephrol.* 2013; 8(7): 1135–42. 10.2215/CJN.09600912 23449770 PMC3700695

[56] Nadim MK Genyk YS Tokin C: Impact of the etiology of acute kidney injury on outcomes following liver transplantation: Acute tubular necrosis versus hepatorenal syndrome. *Liver Transpl.* 2012; 18(5): 539–48. 10.1002/lt.23384 22250075

[57] Patidar KR Kang Le Bajaj JS: Fractional excretion of urea: A simple tool for the differential diagnosis of acute kidney injury in cirrhosis. *Hepatology.* 2018; 68(1): 224–233. 10.1002/hep.29772 29315697 PMC6033653

[58] Alsaad AA Wadei HM: Fractional excretion of sodium in hepatorenal syndrome: Clinical and pathological correlation. *World J Hepatol.* 2016; 8(34): 1497–1501. 10.4254/wjh.v8.i34.1497 28008340 PMC5143430

[59] Belcher JM Sanyal AJ Peixoto AJ: Kidney biomarkers and differential diagnosis of patients with cirrhosis and acute kidney injury. *Hepatology.* 2014; 60(2): 622–32. 10.1002/hep.26980 24375576 PMC4065642

[60] Ariza X Solà E Elia C: Analysis of a urinary biomarker panel for clinical outcomes assessment in cirrhosis. *PLoS One.* 2015; 10(6): e0128145. 10.1371/journal.pone.0128145 26042740 PMC4456079

[61] Huelin P Solà E Elia C: Neutrophil Gelatinase-Associated Lipocalin for Assessment of Acute Kidney Injury in Cirrhosis: A Prospective Study. *Hepatology.* 2019; 70(1): 319–33. 10.1002/hep.30592 30810244

[62] Mandorfer M Bota S Schwabl P: Nonselective β blockers increase risk for hepatorenal syndrome and death in patients with cirrhosis and spontaneous bacterial peritonitis. *Gastroenterology.* 2014; 146(7): 1680–90.e1. 10.1053/j.gastro.2014.03.005 24631577

[63] Krag A Bendtsen F Henriksen JH: Low cardiac output predicts development of hepatorenal syndrome and survival in patients with cirrhosis and ascites. *Gut.* 2010; 59(1): 105–10. 10.1136/gut.2009.180570 19837678

[64] Reiberger T Mandorfer M: Beta adrenergic blockade and decompensated cirrhosis. *J Hepatol.* 2017; 66(4): 849–59. 10.1016/j.jhep.2016.11.001 27864004

[65] Mandorfer M Reiberger T: Beta blockers and cirrhosis, 2016. *Dig Liver Dis.* 2017; 49(1): 3–10. 10.1016/j.dld.2016.09.013 27717792

[66] Huelin P Piano S Solà E: Validation of a Staging System for Acute Kidney Injury in Patients With Cirrhosis and Association With Acute-on-Chronic Liver Failure. *Clin Gastroenterol Hepatol.* 2017; 15(3): 438–445.e5. 10.1016/j.cgh.2016.09.156 27720915

[67] Bernardi M Angeli P Claria J: Albumin in decompensated cirrhosis: New concepts and perspectives. *Gut.* 2020; 69(6): 1127–38. 10.1136/gutjnl-2019-318843 32102926 PMC7282556

[68] Fernández J Clària J Amorós A: Effects of Albumin Treatment on Systemic and Portal Hemodynamics and Systemic Inflammation in Patients With Decompensated Cirrhosis. *Gastroenterology.* 2019; 157(1): 149–62. 10.1053/j.gastro.2019.03.021 30905652

[69] Bortoluzzi A Ceolotto G Gola E: Positive cardiac inotropic effect of albumin infusion in rodents with cirrhosis and ascites: Molecular mechanisms. *Hepatology.* 2013; 57(1): 266–76. 10.1002/hep.26021 22911662

[70] China L Maini A Skene SS: Albumin Counteracts Immune-Suppressive Effects of Lipid Mediators in Patients With Advanced Liver Disease. *Clin Gastroenterol Hepatol.* 2018; 16(5): 738–747.e7. 10.1016/j.cgh.2017.08.027 28859868 PMC6168974

[71] Garcia-Martinez R Noiret L Sen S: Albumin infusion improves renal blood flow autoregulation in patients with acute decompensation of cirrhosis and acute kidney injury. *Liver Int.* 2015; 35(2): 335–43. 10.1111/liv.12528 24620819

[72] Ginès P Titó L Arroyo V: Randomized comparative study of therapeutic paracentesis with and without intravenous albumin in cirrhosis. *Gastroenterology.* 1988; 94(6): 1493–502. 10.1016/0016-5085(88)90691-9 3360270

[73] Luca A García-Pagán JC Bosch J: Beneficial effects of intravenous albumin infusion on the hemodynamic and humoral changes after total paracentesis. *Hepatology.* 1995; 22(3): 753–8. 10.1002/hep.1840220310 7657279

[74] Bernardi M Caraceni P Navickis RJ: Albumin infusion in patients undergoing large-volume paracentesis: A meta-analysis of randomized trials. *Hepatology.* 2012; 55(4): 1172–81. 10.1002/hep.24786 22095893

[75] Gines A Fernandez-Esparrach G Monescillo A: Randomized trial comparing albumin, dextran 70, and polygeline in cirrhotic patients with ascites treated by paracentesis. *Gastroenterology.* 1996; 111(4): 1002–10. 10.1016/s0016-5085(96)70068-9 8831595

[76] Sort P Navasa M Arroyo V: Effect of intravenous albumin on renal impairment and mortality in patients with cirrhosis and spontaneous bacterial peritonitis. *N Engl J Med.* 1999; 341(6): 403–9. 10.1056/NEJM199908053410603 10432325

[77] Fernández J Monteagudo J Bargallo X: A randomized unblinded pilot study comparing albumin versus hydroxyethyl starch in spontaneous bacterial peritonitis. *Hepatology.* 2005; 42(3): 627–34. 10.1002/hep.20829 16108036

[78] Ortega R Ginès P Uriz J: Terlipressin therapy with and without albumin for patients with hepatorenal syndrome: Results of a prospective, nonrandomized study. *Hepatology.* 2002; 36(4 Pt 1): 941–8. 10.1053/jhep.2002.35819 12297842

[79] Caraceni P Riggio O Angeli P: Long-term albumin administration in decompensated cirrhosis (ANSWER): An open-label randomised trial. *Lancet.* 2018; 391(10138): 2417–29. 10.1016/S0140-6736(18)30840-7 29861076

[80] Di Pascoli M Fasolato S Piano S: Long-term administration of human albumin improves survival in patients with cirrhosis and refractory ascites. *Liver Int.* 2019; 39(1): 98–105. 10.1111/liv.13968 30230204

[81] Solà E Solé C Simón-Talero M: Midodrine and albumin for prevention of complications in patients with cirrhosis awaiting liver transplantation. A randomized placebo-controlled trial. *J Hepatol.* 2018; 69(6): 1250–9. 10.1016/j.jhep.2018.08.006 30138685

[82] China L Freemantle N Forrest E: A Randomized Trial of Albumin Infusions in Hospitalized Patients with Cirrhosis. *N Engl J Med.* 2021; 384(9): 808–17. 10.1056/NEJMoa2022166 33657293

[83] Thévenot T Bureau C Oberti F: Effect of albumin in cirrhotic patients with infection other than spontaneous bacterial peritonitis. A randomized trial. *J Hepatol.* 2015; 62(4): 822–30. 10.1016/j.jhep.2014.11.017 25463545

[84] Fernández J Angeli P Trebicka J: Efficacy of Albumin Treatment for Patients with Cirrhosis and Infections Unrelated to Spontaneous Bacterial Peritonitis. *Clin Gastroenterol Hepatol.* 2020; 18(4): 963–973.e14. 10.1016/j.cgh.2019.07.055 31394283

[85] Guevara M Terra C Nazar A: Albumin for bacterial infections other than spontaneous bacterial peritonitis in cirrhosis. A randomized, controlled study. *J Hepatol.* 2012; 57(4): 759–65. 10.1016/j.jhep.2012.06.013 22732511

[86] Velez JCQ Nietert PJ: Therapeutic response to vasoconstrictors in hepatorenal syndrome parallels increase in mean arterial pressure: A pooled analysis of clinical trials. *Am J Kidney Dis.* 2011; 58(6): 928–38. 10.1053/j.ajkd.2011.07.017 21962618 PMC3251915

[87] Facciorusso A Chandar AK Murad MH: Comparative efficacy of pharmacological strategies for management of type 1 hepatorenal syndrome: A systematic review and network meta-analysis. *Lancet Gastroenterol Hepatol.* 2017; 2(2): 94–102. 10.1016/S2468-1253(16)30157-1 28403995

[88] Jamil K Pappas SC Devarakonda KR: In vitro binding and receptor-mediated activity of terlipressin at vasopressin receptors V_1_ and V_2_. *J Exp Pharmacol.* 2017; 10: 1–7. 10.2147/JEP.S146034 29302194 PMC5741980

[89] Tanoue A Ito S Honda K: The vasopressin V1b receptor critically regulates hypothalamic-pituitary-adrenal axis activity under both stress and resting conditions. *J Clin Invest.* 2004; 113(2): 302–9. 10.1172/JCI19656 14722621 PMC311433

[90] Bui TNN Sandar S Luna G: An investigation of reconstituted terlipressin infusion stability for use in hepatorenal syndrome. *Sci Rep.* 2020; 10(1): 21037. 10.1038/s41598-020-78044-4 33273555 PMC7712657

[91] Cavallin M Piano S Romano A: Terlipressin given by continuous intravenous infusion versus intravenous boluses in the treatment of hepatorenal syndrome: A randomized controlled study. *Hepatology.* 2016; 63(3): 983–92. 10.1002/hep.28396 26659927

[92] Reiberger T Püspök A Schoder M: Austrian consensus guidelines on the management and treatment of portal hypertension (Billroth III). *Wien Klin Wochenschr.* 2017; 129(Suppl 3): 135–58. 10.1007/s00508-017-1262-3 29063233 PMC5674135

[93] Cavallin M Kamath PS Merli M: Terlipressin plus albumin versus midodrine and octreotide plus albumin in the treatment of hepatorenal syndrome: A randomized trial. *Hepatology.* 2015; 62(2): 567–74. 10.1002/hep.27709 25644760

[94] Best LM Freeman SC Sutton AJ: Treatment for hepatorenal syndrome in people with decompensated liver cirrhosis: A network meta-analysis. *Cochrane Database Syst Rev.* 2019; 9(9): CD013103. 10.1002/14651858.CD013103.pub2 31513287 PMC6740336

[95] Moreau R Durand F Poynard T: Terlipressin in patients with cirrhosis and type 1 hepatorenal syndrome: A retrospective multicenter study. *Gastroenterology.* 2002; 122(4): 923–30. 10.1053/gast.2002.32364 11910344

[96] Sanyal AJ Boyer T Garcia-Tsao G: A randomized, prospective, double-blind, placebo-controlled trial of terlipressin for type 1 hepatorenal syndrome. *Gastroenterology.* 2008; 134(5): 1360–8. 10.1053/j.gastro.2008.02.014 18471513 PMC3730280

[97] Boyer TD Sanyal AJ Wong F: Terlipressin Plus Albumin Is More Effective Than Albumin Alone in Improving Renal Function in Patients With Cirrhosis and Hepatorenal Syndrome Type 1. *Gastroenterology.* 2016; 150(7): 1579–1589.e2. 10.1053/j.gastro.2016.02.026 26896734

[98] Rodríguez E Elia C Solà E: Terlipressin and albumin for type-1 hepatorenal syndrome associated with sepsis. *J Hepatol.* 2014; 60(5): 955–61. 10.1016/j.jhep.2013.12.032 24447876

[99] Duvoux C Zanditenas D Hézode C: Effects of noradrenalin and albumin in patients with type I hepatorenal syndrome: A pilot study. *Hepatology.* 2002; 36(2): 374–80. 10.1053/jhep.2002.34343 12143045

[100] Alessandria C Ottobrelli A Debernardi-Venon W: Noradrenalin vs terlipressin in patients with hepatorenal syndrome: A prospective, randomized, unblinded, pilot study. *J Hepatol.* 2007; 47(4): 499–505. 10.1016/j.jhep.2007.04.010 17560680

[101] Singh V Ghosh S Singh B: Noradrenaline vs. terlipressin in the treatment of hepatorenal syndrome: A randomized study. *J Hepatol.* 2012; 56(6): 1293–8. 10.1016/j.jhep.2012.01.012 22322237

[102] Gifford FJ Morling JR Fallowfield JA: Systematic review with meta-analysis: Vasoactive drugs for the treatment of hepatorenal syndrome type 1. *Aliment Pharmacol Ther.* 2017; 45(5): 593–603. 10.1111/apt.13912 28052382

[103] Piano S Schmidt HH Ariza X: Association Between Grade of Acute on Chronic Liver Failure and Response to Terlipressin and Albumin in Patients With Hepatorenal Syndrome. *Clin Gastroenterol Hepatol.* 2018; 16(11): 1792–1800.e3. 10.1016/j.cgh.2018.01.035 29391267

[104] Wong F Pappas SC Boyer TD: Terlipressin Improves Renal Function and Reverses Hepatorenal Syndrome in Patients With Systemic Inflammatory Response Syndrome. *Clin Gastroenterol Hepatol.* 2017; 15(2): 266–272.e1. 10.1016/j.cgh.2016.07.016 27464593

[105] Sanyal AJ Boyer TD Frederick RT: Reversal of hepatorenal syndrome type 1 with terlipressin plus albumin vs. placebo plus albumin in a pooled analysis of the OT-0401 and REVERSE randomised clinical studies. *Aliment Pharmacol Ther.* 2017; 45(11): 1390–402. 10.1111/apt.14052 28370090 PMC5434950

[106] Wong F Pappas SC Curry MP: Terlipressin plus Albumin for the Treatment of Type 1 Hepatorenal Syndrome. *N Engl J Med.* 2021; 384(9): 818–28. 10.1056/NEJMoa2008290 33657294

[107] Arora V Maiwall R Rajan V: Terlipressin Is Superior to Noradrenaline in the Management of Acute Kidney Injury in Acute on Chronic Liver Failure. *Hepatology.* 2020; 71(2): 600–10. 10.1002/hep.30208 30076614

[108] Facciorusso A Antonino M Orsitto E: Primary and secondary prophylaxis of spontaneous bacterial peritonitis: Current state of the art. *Expert Rev Gastroenterol Hepatol.* 2019; 13(8): 751–9. 10.1080/17474124.2019.1644167 31304804

[109] Fernández J Navasa M Planas R: Primary prophylaxis of spontaneous bacterial peritonitis delays hepatorenal syndrome and improves survival in cirrhosis. *Gastroenterology.* 2007; 133(3): 818–24. 10.1053/j.gastro.2007.06.065 17854593

[110] Moreau R Elkrief L Bureau C: Effects of Long-term Norfloxacin Therapy in Patients With Advanced Cirrhosis. *Gastroenterology.* 2018; 155(6): 1816–1827.e9. 10.1053/j.gastro.2018.08.026 30144431

[111] Piano S Singh V Caraceni P: Epidemiology and Effects of Bacterial Infections in Patients With Cirrhosis Worldwide. *Gastroenterology.* 2019; 156(5): 1368–1380.e10. 10.1053/j.gastro.2018.12.005 30552895

[112] García-Pagán JC Saffo S Mandorfer M: Where does TIPS fit in the management of patients with cirrhosis? *JHEP Rep.* 2020; 2(4): 100122. 10.1016/j.jhepr.2020.100122 32671331 PMC7347999

[113] Turco L Villanueva C La Mura V: Lowering Portal Pressure Improves Outcomes of Patients With Cirrhosis, With or Without Ascites: A Meta-Analysis. *Clin Gastroenterol Hepatol.* 2020; 18(2): 313–327.e6. 10.1016/j.cgh.2019.05.050 31176013

[114] Mandorfer M Hernández-Gea V Reiberger T: Hepatic Venous Pressure Gradient Response in Non-Selective Beta-Blocker Treatment—Is It Worth Measuring? *Curr Hepatology Rep.* 2019; 18: 174–86. 10.1007/s11901-019-00469-x

[115] Guevara M Ginès P Bandi JC: Transjugular intrahepatic portosystemic shunt in hepatorenal syndrome: Effects on renal function and vasoactive systems. *Hepatology.* 1998; 28(2): 416–22. 10.1002/hep.510280219 9696006

[116] Ginès P Uriz J Calahorra B: Transjugular intrahepatic portosystemic shunting versus paracentesis plus albumin for refractory ascites in cirrhosis. *Gastroenterology.* 2002; 123(6): 1839–47. 10.1053/gast.2002.37073 12454841

[117] Testino G Ferro C Sumberaz A: Type-2 hepatorenal syndrome and refractory ascites: Role of transjugular intrahepatic portosystemic stent-shunt in eighteen patients with advanced cirrhosis awaiting orthotopic liver transplantation. *Hepatogastroenterology.* 2003; 50(54): 1753–5. 14696397

[118] Bureau C Thabut D Oberti F: Transjugular Intrahepatic Portosystemic Shunts With Covered Stents Increase Transplant-Free Survival of Patients With Cirrhosis and Recurrent Ascites. *Gastroenterology.* 2017; 152(1): 157–63. 10.1053/j.gastro.2016.09.016 27663604

[119] Wong F Pantea L Sniderman K: Midodrine, octreotide, albumin, and TIPS in selected patients with cirrhosis and type 1 hepatorenal syndrome. *Hepatology.* 2004; 40(1): 55–64. 10.1002/hep.20262 15239086

[120] Bosch J: Small diameter shunts should lead to safe expansion of the use of TIPS. *J Hepatol.* 2021; 74(1): 230–4. 10.1016/j.jhep.2020.09.018 32987029

[121] Praktiknjo M Abu-Omar J Chang J: Controlled underdilation using novel Viatorr controlled expansion stents improves survival after transjugular intrahepatic portosystemic shunt implantation. *JHEP Rep.* 2021; 63(3): 100264. 10.1016/j.jhepr.2021.100264 PMC8113713 34013182

[122] Schepis F Vizzutti F Garcia-Tsao G: Under-dilated TIPS Associate With Efficacy and Reduced Encephalopathy in a Prospective, Non-randomized Study of Patients With Cirrhosis. *Clin Gastroenterol Hepatol.* 2018; 16(7): 1153–1162.e7. 10.1016/j.cgh.2018.01.029 29378312

[123] Wang Q Lv Y Bai M: Eight millimetre covered TIPS does not compromise shunt function but reduces hepatic encephalopathy in preventing variceal rebleeding. * J Hepatol.* 2017; 67(3): 508–16. 10.1016/j.jhep.2017.05.006 28506905

[124] Lenhart A Hussain S Salgia R: Chances of Renal Recovery or Liver Transplantation After Hospitalization for Alcoholic Liver Disease Requiring Dialysis. *Dig Dis Sci.* 2018; 63(10): 2800–9. 10.1007/s10620-018-5170-9 29934721

[125] Zhang Z Maddukuri G Jaipaul N: Role of renal replacement therapy in patients with type 1 hepatorenal syndrome receiving combination treatment of vasoconstrictor plus albumin. *J Crit Care.* 2015; 30(5): 969–74. 10.1016/j.jcrc.2015.05.006 26051980

[126] Fraley DS Burr R Bernardini J: Impact of acute renal failure on mortality in end-stage liver disease with or without transplantation. *Kidney Int.* 1998; 54(2): 518–24. 10.1046/j.1523-1755.1998.00004.x 9690218

[127] Wong LP Blackley MP Andreoni KA: Survival of liver transplant candidates with acute renal failure receiving renal replacement therapy. *Kidney Int.* 2005; 68(1): 362–70. 10.1111/j.1523-1755.2005.00408.x 15954928

[128] Allegretti AS Parada XV Eneanya ND: Prognosis of Patients with Cirrhosis and AKI Who Initiate RRT. *Clin J Am Soc Nephrol.* 2018; 13(1): 16–25. 10.2215/CJN.03610417 29122911 PMC5753306

[129] Piano S Gambino C Vettore E: Response to Terlipressin and Albumin Is Associated With Improved Liver Transplant Outcomes in Patients With Hepatorenal Syndrome. *Hepatology.* 2020; 73(5): 1909–1919. 10.1002/hep.31529 32870499

[130] Rodriguez E Henrique Pereira G Solà E: Treatment of type 2 hepatorenal syndrome in patients awaiting transplantation: Effects on kidney function and transplantation outcomes. *Liver Transpl.* 2015; 21(11): 1347–54. 10.1002/lt.24210 26178066

[131] Northup PG Argo CK Bakhru MR: Pretransplant predictors of recovery of renal function after liver transplantation. *Liver Transpl.* 2010; 16(4): 440–6. 10.1002/lt.22008 20205164

[132] Nadim MK Sung RS Davis CL: Simultaneous liver-kidney transplantation summit: Current state and future directions. *Am J Transplant.* 2012; 12(11): 2901–8. 10.1111/j.1600-6143.2012.04190.x 22822723

[133] Ruiz R Barri YM Jennings LW: Hepatorenal syndrome: A proposal for kidney after liver transplantation (KALT). *Liver Transpl.* 2007; 13(6): 838–43. 10.1002/lt.21149 17539003

[134] Paramesh AS Roayaie S Doan Y: Post-liver transplant acute renal failure: Factors predicting development of end-stage renal disease. *Clin Transplant.* 2004; 18(1): 94–9. 10.1046/j.1399-0012.2003.00132.x 15108777

